# Restoring nature with microbes: bioremediation in the world’s biodiversity hotspots

**DOI:** 10.1128/aem.01442-25

**Published:** 2025-09-09

**Authors:** Laura T. Morales-Mancera, Laura L. Diaz Ortiz, Jaime E. Gutiérrez-Fonseca, Martha J. Vives

**Affiliations:** 1 Department of Biological Sciences, Centro de Investigaciones Microbiológicas, Universidad de los Andes 27991, Bogotá, Colombia; Michigan State University, East Lansing, Michigan, USA

**Keywords:** bioremediation, microbial remediation, megadiverse countries, biodiversity, waste water, hydrocarbon contamination

## Abstract

Megadiverse countries, which collectively harbor over 70% of the planet’s terrestrial biodiversity, play a crucial role in global conservation efforts. However, many of these nations, primarily in the developing world, face significant environmental challenges that threaten biodiversity, including pollution, habitat loss, and climate change. Among these issues, pollution—driven by industrialization, agriculture, and improper waste disposal—has emerged as a critical concern, particularly for water and soil ecosystems. Bioremediation, a biological approach to mitigate environmental pollution, has gained prominence as a sustainable and cost-effective alternative to conventional physicochemical methods. This study explores microbial bioremediation research and scales up in megadiverse developing countries, focusing on hydrocarbon and wastewater pollution. Here, we conduct a meta-analysis of the literature and examine the underlying factors that contribute to disparities in the development and implementation of bioremediation initiatives. Given the growing urgency of pollution control in biodiversity-rich regions, understanding the role of microbial communities in bioremediation is essential. By leveraging biological solutions, megadiverse nations can address pollution challenges while preserving their unique ecosystems. This review highlights existing bioremediation practices, their successes, and the potential for further implementation to safeguard both environmental and human health.

## MEGADIVERSE COUNTRIES

Currently, there are 17 countries on the list of megadiverse countries also known as the Like-Minded Megadiverse Countries: Brazil, Colombia, Mexico, Perú, Ecuador, the United States, Australia, China, Indonesia, Papua New Guinea, the Democratic Republic of Congo, South Africa, Madagascar, Malaysia, Venezuela, the Philippines, and India. These countries harbor the highest levels of biodiversity and numerous endemic species and are committed to safeguarding and prioritizing the preservation and sustainable use of the world’s biological diversity. This concept, proposed by Russell Mittermeier in 1988, is used to promote the protection of natural biodiversity. Megadiverse countries contain at least 70% of the planet’s terrestrial biodiversity, including most non-aquatic vertebrate and higher plant species, despite covering only about 10% of Earth’s surface (1).

Biodiversity is declining, emphasizing the need to address its key drivers: climate change, land- and sea-use changes, resource exploitation, invasive species, and pollution. These factors impact ecosystems differently across continents (2).

Pollution, primarily from human activities like industrialization, mining, and agriculture, harms both the environment and human health (3). These issues are more common in developing countries, due to waste mismanagement which exacerbates environmental issues (4). Noteworthy, most of the megadiverse countries—except for Australia and the United States—are developing nations (Organization for Economic Co-operation and Development, 2021), where environmental problems are more severe.

## OVERVIEW OF BIOREMEDIATION: APPROACHES AND APPLICATIONS

Water, soil, and air pollutions pose significant global risks to ecosystems and human health, primarily due to human activities that release toxic compounds, including organic dyes, pesticides, hydrocarbons, inorganic compounds, and heavy metals. Many of these pollutants are hazardous due to their carcinogenicity, persistence, and non-biodegradability (5). For instance, petroleum hydrocarbons negatively impact aquatic communities by adsorbing particulate matter, persisting in sediments, and generating toxic effects across trophic levels (6, 7). Additionally, hydrocarbon layers on water surfaces reduce light penetration, photosynthesis, and oxygen dissolution, further harming ecosystems (8).

Various physicochemical techniques have been used to remove pollutants from water, including chemical precipitation, ion exchange, electrodialysis, ultrafiltration, and reverse osmosis (9). However, bioremediation has gained attention for its cost-effectiveness and sustainability. This approach includes methods like composting, bioreactors, bioaugmentation, biostimulation, and phyto/phycoremediation, applicable to water, soil, and, less commonly, air (10).

Microbial bioremediation utilizes microorganisms to degrade pollutants into less harmful compounds (CO_2_, CH_4_, H_2_O, and biomass) without damaging the environment. Many microorganisms naturally possess the ability to degrade toxic substances, which makes them ideal candidates for bioremediation (11, 12). In nature, microorganisms are primarily found in consortia: a natural association of two or more microbial populations of different species that act together as a community in a complex system, where all species benefit from each other, often exhibiting synergistic or syntrophic lifestyles, enhancing the efficiency of pollutant degradation (13). Given its potential, bioremediation is a promising strategy for mitigating pollution. In this study, we reviewed the microbial bioremediation experiences from megadiverse developing countries. Heavy metals contamination is a major issue that has been thoroughly addressed in recent literature (14, 15). Hence, we will focus on hydrocarbons and wastewater pollution.

## STATE OF THE ART IN MICROBIAL BIOREMEDIATION

Table 1 presents the microorganisms most commonly reported in hydrocarbon and wastewater bioremediation. Several bacterial genera, including *Pseudomonas*, *Bacillus*, *Halomonas*, *Vibrio*, and *Enterobacter*, have been widely studied for hydrocarbon degradation but are also recognized as human pathogens, posing risks in open bioremediation contexts. Similarly, fungal genera such as *Aspergillus*, *Fusarium*, *Phanerochaete*, and *Irpex* are extensively studied for polycyclic aromatic hydrocarbon (PAH) degradation but include pathogenic species. In contrast, microalgae and cyanobacteria play a significant role in wastewater decontamination, with pathogenic species being rare, making them a safer alternative for *in situ* bioremediation in natural water bodies.

**TABLE 1:** Common microorganisms in bioremediation studies, pollutants, and known microbial pathogenicity

Organism	Genus	Most common species reported in bioremediation	Pollutant	Removal/degradation^*a*^	Pathogenicity^*b*^
Bacteria	*Alcaligenes*	*Alcaligenes aquatilis*	Crude oil	Degradation (16)	No
	*Bacillus*	*Bacillus subtilis*	Petroleum hydrocarbon	Degradation (17)	H (uncommon) (18–23)
	*Enterobacter*	*Enterobacter* sp.	Diesel	Degradation (24)	H (frequently) (25–30)
	*Flavobacterium*	*Flavobacterium petrolei*	Diesel	Degradation (31)	No
	*Pseudomonas*	*P. aeruginosa*	Phenanthrene, pyrene, hexadecane	Degradation (32, 33)	H and A (frequently) (34–38)
		*Pseudomonas pseudoalcaligenes*	Phenanthrene and pyrene	Degradation (39)	H (uncommon) (40–42)
	*Alcanivorax*	*Alcanivorax* sp.	Alkanes	Degradation (43, 44)	No
	*Halomonas*	*Halomonas* sp.	Hexadecane	Degradation (45)	H (uncommon) (46–49)
		*Halomonas pacifica*	Naphthalene	Degradation (50)	H (uncommon) (51)
	*Ralstonia*	*Ralstonia pickettii*	Crude oil	Degradation (52)	H (uncommon) (53–58)
	*Acinetobacter*	*Acinetobacter* sp.	Aliphatic alkanes	Degradation (59)	H (frequently) (60–66)
	*Achromobacter*	*Achromobacter insolitus*	Polyaromatic	Degradation (67)	H (uncommon) (68)
	*Ochrobactrum*	*Ochrobactrum anthropi*	Crude oil	Degradation (69)	H (frequently) (70–75)
	*Stenotrophomonas*	*Stenotrophomonas* sp.	PAHs	Degradation (76)	H (uncommon) (77–79)
	*Vibrio*	*Vibrio* sp.	PAHs	Degradation (80)	H and A (frequently) (80–86)
Cyanobacteria	*Spirulina*	*Spirulina platensis*	Waste-activated sludge	Degradation (87)	No
	*Oscillatoria*	*Oscillatoria animalis*	Wastewater	Removal (88)	No
	*Synechococcus*	*Synechococcus elongatus*	Wastewater	Removal (89)	No
	*Nostoc*	*Nostoc muscorum*	Sewage and industrial wastewater	Removal (90)	No
	*Anabaena*	*Anabaena subcylinderica*	Sewage and industrial wastewater	Removal (90)	No
Fungi	*Aspergillus*	*Aspergillus* sp.	Polycyclic aromatics, 2–7 rings	Degradation (91)	H and A (frequently) (92–98)
	*Fusarium*	*Fusarium oxysporum*	Polycyclic aromatics, 2–3 rings	Degradation (91)	H, A, and P (frequently) (99–104)
	*Pleurotus*	*Pleurotus ostreatus*	Naphthalene, anthracene, acenaphthene, pyrene, fluorene	Degradation (105)	No
	*Phanerochaete*	*Phanerochaete chrysosporium*	Anthracene	Degradation (106)	H (uncommon) (107)
	*Irpex*	*Irpex lacteus*	Anthracene, phenanthrene, fluoroanthene, pyrene	Degradation (108)	H (uncommon) (109, 110)
	*Scedosporium*	*S. apiospermum*	Toluene	Degradation (111)	H (uncommon) (112–117)
Microalgae	*Chlorella*	*Chlorella vulgaris*	PAHs	Removal (118)	No
	*Scenedesmus*	*Scenedesmus quadricauda*	PAHs	Removal (118)	No
		*Scenedesmus platydiscus*	PAHs	Removal (118)	No
		*Scenedesmus bijugatus*	Pesticides	Removal (118)	No
	*Chlamydomonas*	*Chlamydomonas* sp.	Industrial effluent	Removal (119)	No
	*Nannochloropsis*	*Nannochloropsis oculate*	Hydrocarbons	Removal (120)	No

^*a*^ Removal refers to the complete or partial elimination of the pollutant, as degradation refers to the breakdown of the compounds. Here, removal and degradation are reported as the original publication did.

^*b*^ Pathogenicity refers specifically to species, not to the genus. No, not reported; H, in humans; A, in other animals; P, in plants.

Microorganisms in bioremediation have been studied both individually and within natural or artificial consortia (Table 2). Consortia have several advantages: (i) they can perform complex functions that populations of individual species could not achieve; (ii) living in association can provide greater resistance to environmental fluctuations, and (iii) they promote the stability of its members over time (121). Bioremediation consortia can be defined (with known species) or undefined (naturally occurring in contaminated sites). A drawback of defined consortia is the need for multiple strains with diverse degradative properties, and since these strains are not accustomed to living in association, toxic substances may be produced for some members of the consortium (122). Biodegrading consortia can include bacteria, fungi, protists, microalgae, and plants and are capable of degrading organic pollutants like synthetic dyes, PAHs, and polychlorinated biphenyls, with the majority represented by bacterial, fungal, and microalgae species (12).

**TABLE 2:** Examples of consortia reported in hydrocarbons and wastewater bioremediation assays

Microorganisms in consortia	Species	Pollutants	Reference
Fungi/bacteria	*Phellinus* sp. and *Bacillus pumilus*	Naphthalene, anthracene, acenaphthene, pyrene, and fluorene	(123)
	*Polyporus sulphureus* and *B. pumilus*		
	*Pycnoporus sanguineus* and *Pseudomonas* sp.		
	*Coriolus versicolor* and *Pseudomonas* sp.		
	*Pleurotus ostreatus* and *Pseudomonas* sp.		
	*Fomitopsis palustris* and *Pseudomonas* sp.		
	*Daedalea elegans* and *Pseudomonas* sp.		
Microalgae/bacteria	*Selenastrum capricornutum* and *Mycobacterium* sp.	Pyrene	(124)
	*Tetradesmus obliquus* and *Variovorax paradoxus*	Dairy farm wastewater and poultry slaughterhouse wastewater	(125)
	*Chlorella sorokiniana, Chlorella* sp.*, Klebsiella pneumoniae,* and *Acinetobacter calcoaceticus*.	Artificial wastewater	(126)
Microalgae/bacteria/archaea.	*Chlorella pyrenoidosa* and activated sludge	Swine wastewater	(127)
	*C. sorokiniana* and activated sludge	Synthetic wastewater	(128)
Microalgae/fungi	*Chlorella* sp. and *Penicillium* sp.	Wastewater	(129)
	*C. pyrenoidosa* and *Debaryomyces hansenii*	Synthetic wastewater	(130)
Microalgae/microalgae	*Ulothrix zonata*, *Ulothrix aequalis*, *Rhizoclonium hieroglyphicum*, and *Oedogonium* sp.	Manure wastewater	(131)
	*Chlamydomonas reinhardtii, Scenedesmus rubescens*, and *Chlorella vulgaris*	Municipal wastewater	(132)
	*Chlorella* sp. and *Scenedesmus* sp.	Municipal wastewater	(133)
	*Chlorella* sp., *Scenedesmus* sp., *Chlorococcum* sp. and *Chroococcus* sp.	Sewage water	(134)
	*Chlorococcum* sp., *Scenedesmus* sp., *Chlorella* sp., and *Phaeodactylum tricornutum*	Synthetic municipal wastewater	(135)

Recent research has increasingly focused on microalgae-bacteria consortia for hydrocarbon degradation (136–139) and wastewater treatment (140–142). These consortia are effective in removing various organic contaminants due to the unique metabolic capabilities of each microorganism (143). Additionally, bacteria-microalgae interactions enhance nutrient removal from wastewater and boost biomass production, leading to valuable byproducts (144).

## BIOREMEDIATION IN MEGADIVERSE COUNTRIES

Hydrocarbon contamination and wastewater pollution are among the most pressing environmental challenges in megadiverse countries. Oil remains a vital energy source for both developed and developing nations, producing fuels and a variety of industrial raw materials (145). However, its production and use result in significant environmental harm, contaminating soil, water, and air through oil spills, toxic waste, and greenhouse gas emissions (146).

Petrochemical wastewater, often containing hydrocarbons, heavy metals, and persistent organic compounds, ranks among the most hazardous pollutants due to its low biodegradability and high toxicity, which limit the effectiveness of conventional treatment methods (147). Oil spills, whether caused by natural disasters or human actions—including deliberate attacks on oil infrastructure in developing nations—release toxic and carcinogenic hydrocarbons that are resistant to degradation (148). Traditional physicochemical methods for removing or degrading hydrocarbons are often inadequate and may cause further environmental damage (149), making bioremediation an increasingly preferred and sustainable alternative (150).

Water pollution represents another major global issue, with particularly severe implications in megadiverse countries, where freshwater ecosystems are critical to biodiversity conservation (151). These ecosystems are highly vulnerable to contamination from untreated wastewater, posing risks to both aquatic life and nearby human communities. In countries like Brazil, Colombia, Mexico, China, and India, water bodies rich in endemic species are frequently exposed to inadequately treated wastewater from industrial, agricultural, and urban sources. This pollution degrades ecosystems and endangers communities reliant on these water sources for food, water, and economic activities (152, 153).

Residential wastewater is a major contributor to water pollution in urbanized regions of Latin America (LA), where data on the wastewater sector remain limited despite high population density (154–156). Treatment systems in the region face challenges such as weak legislation, limited resource recovery policies, and infrastructure-focused financing models (156, 157). Brazil’s approach—prioritizing receiving water quality over effluent standards—has influenced broader wastewater policies across LA and the Caribbean (158). Conventional wastewater treatment plants, including stabilization ponds and activated sludge systems, are predominant, but small-scale facilities frequently struggle with inefficiencies and compliance issues (159–161).

Bioremediation stands out as a cost-effective and environmentally friendly solution, particularly suitable for developing countries. To explore its implementation, we conducted a systematic review of bioremediation studies carried out in eight megadiverse countries: Brazil, Colombia, Mexico, Peru, Ecuador, China, Venezuela, and India. These countries were selected based on economic relevance and data availability. Databases searched were Science Direct, SCIELO, PubMed, and Google Scholar, using the following search criteria: studies published between 2014 and 2024; key words in English and Spanish “Bioremediation, biodegradation, hydrocarbons, and wastewater”; each word was combined with “bacteria, fungi, and microalgae” separately and combined with names of each country. For wastewater, we focused on organic pollutants, textile/cosmetic/tannery/paper/petroleum dyes, nutrients (P, N), and terms like chemical oxygen demand (COD) and biological oxygen demand (BOD5). Reviews and studies on heavy metals, pharmaceuticals, or plastics were excluded.

### Hydrocarbon bioremediation in megadiverse countries

**Fig 1:**
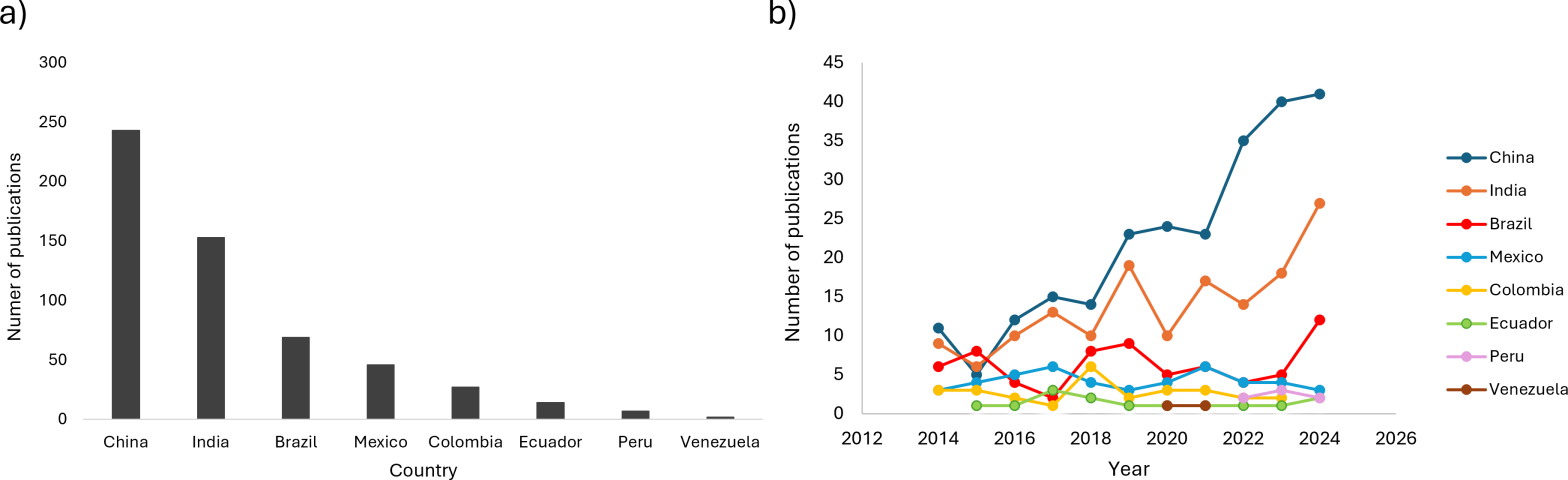
Scientific publications on hydrocarbon bioremediation in megadiverse countries (2014–2024). (**a**) Total publications, (**b**) number of publications for year.

In megadiverse countries, where biodiversity and environmental preservation are critical, multiple bacterial, fungi, cyanobacteria, and microalgae genera have been extensively studied for their role in hydrocarbon bioremediation processes. The systematic search revealed that China is the country with the highest number of scientific publications (160), followed by India and Brazil (with 86 and 62 publications, respectively) (Fig. 1a). These publications include those about the removal and/or degradation of total petroleum hydrocarbons (TPHs), aromatic hydrocarbons, PAHs, and aliphatic hydrocarbons. China plays a key role in global hydrocarbon research (162), driven by the need for land management and pollution remediation, as nearly 4.8 million hectares of land are contaminated by petroleum (163, 164).

Countries with the highest publication numbers have also increased their output in recent years (Fig. 1b). In contrast, Colombia, Peru, Ecuador, Venezuela, and Mexico continue to produce relatively few studies on hydrocarbon removal (Fig. 1b). This low output could be associated with several challenges faced by Latin American researchers, including (i) access to contaminated sites can be challenging (165, 166); (ii) much of the work on bioremediation is carried out by students, who complete their academic programs before completing all the requirements for a publication; hence, very few of these get published; (iii) bioremediation requires long times of experimenting and testing (150), and funding for bioremediation projects is scarce and unsteady. For example, a study focused on scientific production in LA found that investment in research and development across the region remains low compared to regions such as Asia and Europe (167). We consider that these three factors may help explain the low publication production and therefore the limited implementation of bioremediation strategies in the region. A key strategy to overcome these limitations is fostering national and international collaborations. For example, Brazil, the Latin American megadiverse country with the highest number of hydrocarbon bioremediation publications, maintains strong scientific collaborations, particularly with the United States (146).

**Fig 2:**
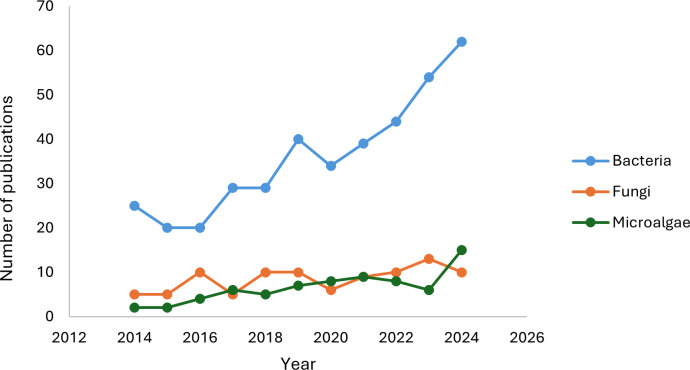
Number of publications on hydrocarbon bioremediation by group of microorganisms in megadiverse countries between 2014 and 2024.

An interesting finding in hydrocarbon bioremediation studies is the predominant use of bacteria over fungi and microalgae in megadiverse countries (Fig. 2). This trend is partly due to the growing research on bacterial biosurfactants, which emulsify hydrocarbons, increasing their solubility and bioavailability (168, 169). Biosurfactants play a crucial role in enhancing petroleum hydrocarbon degradation in aquatic environments (170, 171).

Bacteria efficiently decompose hydrocarbons like saturated alkanes (C15–C35) and PAHs (two to five rings). For instance, a bacterial consortium composed of *Bacillus* sp., *Brevibacillus* sp., and *Acinetobacter* sp. has demonstrated the capacity to achieve degradation rates of 66.32% for saturated hydrocarbons and 63.16% for aromatic hydrocarbons (172, 173).

### Wastewater bioremediation in megadiverse countries

**Fig 3:**
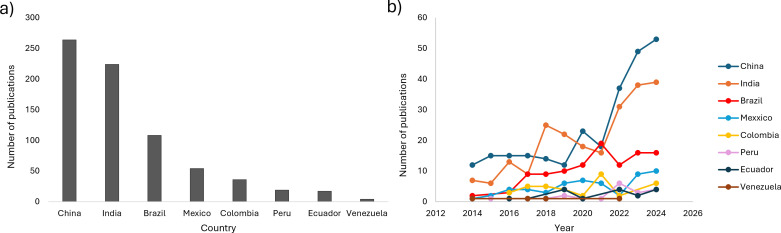
Number of publications about wastewater bioremediation in megadiverse countries (2014–2024). (**a**) Total publications, (**b**) number of publications for year.

Similarly, to the output on hydrocarbon bioremediation, research groups in China, India, and Brazil lead the production of papers on wastewater bioremediation (Fig. 3), and countries such as Colombia, Ecuador, Peru, Venezuela, and Mexico publish fewer papers (Fig. 3). In a review about articles published on wastewater management in 2023, China and India were found among the countries with the highest number of publications on this topic (174). Notably, China and India are at the forefront of research on wastewater bioremediation using microalgae, with systematic reviews highlighting their dominance in this field (175, 176). The highest number of published papers on this subject in 2023 was from China, followed by India, the United States, and Brazil (176).

Wastewater treatment is a key priority due to the scarcity of fresh water and the protection of the environment and human health. There are greater challenges in treating wastewater in developing countries compared to developed countries due to insufficient infrastructure, disorderly and unsustainable urbanization, and the lack of modern technology (152).

Despite the growing development of microorganism-based technologies for industrial pollutant removal in LA, further research and advancement of green strategies remain essential. Sustainable development is crucial for the region, and according to the United Nations Industrial Development Organization, biotechnology plays a key role in this process. LA’s vast biodiversity offers significant potential for alternative technologies that promote sustainability (177).

**Fig 4:**
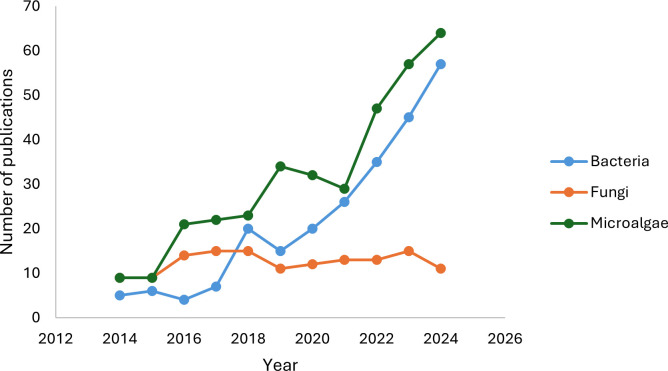
Number of publications on wastewater bioremediation by microorganisms in megadiverse countries between 2014 and 2024.

Unlike hydrocarbon bioremediation studies, wastewater bioremediation in megadiverse countries predominantly utilizes microalgae, followed by bacteria (Fig. 4). Microalgae are ideal for wastewater decontamination due to their rapid growth, CO₂ fixation, high photosynthetic efficiency, adaptability to complex conditions, and ability to consume large amounts of nutrients (178, 179). Additionally, their biomass can be used to produce high-value products such as biodiesel, dyes, aquatic feedstock, and beneficial biopolymers (180, 181).

Despite the widespread use of bacteria and microalgae in both hydrocarbon and wastewater bioremediation, the choice of microorganisms depends on multiple factors. These include using single strains versus microbial consortia; the physicochemical properties of pollutants; environmental conditions such as temperature, pH, salinity, and nutrient availability (182, 183); and the decontamination approach (*in situ* or *ex situ*) (183). Based on these factors, researchers determine whether to employ bacteria, fungi, microalgae, or mixed consortia.

Bioremediation studies are conducted at different scales, with most research occurring at the laboratory level and only a few at pilot or field scales (Table 3). This gap highlights the challenges of translating laboratory findings into scalable applications. Understanding key factors influencing pollutant removal and degradation is crucial, particularly in large-scale bioremediation, where environmental fluctuations and interfering substances can impact efficiency. Small-scale experiments often report higher biodegradation rates compared to field studies (184). For instance, while laboratory conditions demonstrated significant bacterial degradation of phenanthrene and dibenzothiophene, a field-scale experiment under similar conditions showed minimal degradation (185). These differences likely stem from the controlled conditions of laboratory studies, where variables such as temperature, humidity, and nutrient concentrations are more easily regulated, unlike in real-world settings (184, 185).

**TABLE 3:** Published scientific papers reporting either laboratory-scale bioremediation project, pilot-scale experiment, or field scale experiments from 2014 to 2024

Country	Laboratory	Pilot	Field
China	504	13	1
India	372	4	1
Brazil	176	29	3
Mexico	99	7	NRF^*a*^
Colombia	62	11	2
Ecuador	31	1	2
Peru	26	4	NRF
Venezuela	6	NRF	NRF

^*a*^ NRF, no references were found.

Interestingly, Brazil was found to be the country with the highest number of pilot-scale bioremediation studies different from the other countries where this type of studies is rare. This trend can be supported by the fact that the regulation frameworks controlling the use of bioremediation strategies vary from one country to the next. Such variations can be a limitation for the implementation of these studies beyond laboratory scale (186).

Among the critical factors contributing to the gap between laboratory-scale and pilot- or field-scale hydrocarbon and wastewater bioremediation studies is the use of single microorganisms versus microbial consortia. While microbial consortia are employed at all scales, most laboratory studies focus on single strains. This presents challenges when attempting to scale up, as polluted natural sites are highly heterogeneous and contain diverse contaminants. For instance, environments polluted with diesel oil can also contain other contaminants such as motor oils and lubricants (187). In contrast, microbial consortia can metabolize a wider range of pollutants and exhibit cooperative interactions that enhance bioremediation efficiency. For instance, some microorganisms produce compounds that stimulate the growth of others within the consortium, while synergistic relationships and co-metabolism processes promote adaptation to environmental conditions (184). Additionally, consortia that combine different types of microorganisms—such as microalgae and bacteria—have emerged as a cost-effective and efficient alternative for wastewater bioremediation (179).

Other challenges in transferring bioremediation research from the laboratory to the field applications include the limited understanding of microbial behavior in contaminated environments, the variable bioavailability of pollutants, and the lack of interdisciplinary integration when implementing bioremediation strategies in the field (188). Another key aspect during the scaling up process is the reduction in the potential of bioremediation methods. Although these methodologies often demonstrate high potential under controlled laboratory conditions, their performance can decrease at field scale (187). These aspects can be limited when attempting to scale up bioremediation processes from the laboratory to field applications.

Despite these challenges, pilot- and field-scale bioremediation studies have been increasing in recent years. According to the data gathered in this study, both individual microorganisms and microbial consortia are being tested at larger scales more frequently. This trend suggests a growing interest in translating laboratory findings into real-world applications. In some cases, bioremediation even becomes more effective when transitioning from laboratory to pilot- or field-scale. For example, a 2014 study demonstrated that the biodegradation kinetics of TPH at field scale were significantly superior to those observed in laboratory experiments (189), with some estimates suggesting an increase of up to two orders of magnitude (189, 190).

Bioremediation using microalgae has proven more effective when scaled from laboratory to pilot- and field-scale applications in various industrial effluents. In India, several successful pilot projects have demonstrated this potential. At SNAP Natural and Alginate Products in Ranipet, a large-scale facility using *Chroococcus turgidus* achieved complete sludge reduction and pH stabilization at 7.02 and produced valuable by-products such as biofertilizer and aquaculture feed (191). Another study using *Desmococcus olivaceus* in an open raceway pond treated chrome sludge, reducing total dissolved solids (TDS) by 74.24%, phosphate by 86.16%, and chromium by 33.33% (192). Likewise, *Chlorococcum humicola* and *C. turgidus* showed growth in 2.5% sludge from hypochlorite manufacturing, confirming their pilot-scale potential (193). These examples highlight the enhanced performance of phycoremediation at larger scales due to real-world conditions and process optimization.

However, despite the promise of bioremediation, major challenges remain. In many developing countries, research on wastewater bioremediation is often underfunded and unpublished, leading to gaps in knowledge dissemination. The same financial and logistical barriers that limit the publication of hydrocarbon bioremediation studies also affect wastewater treatment research, further hindering the implementation of sustainable solutions.

## BIOREMEDIATION IN COLOMBIA

We selected Colombia for in-depth analysis due to its exceptional biodiversity. Among megadiverse countries, it ranks among the top 5 globally in species richness for birds, amphibians, freshwater fish, butterflies, reptiles, plants, and mammals. However, over 1,000 species are currently threatened by human activities (194, 195). According to national ecosystem mapping, Colombia hosts 98 ecosystem types and over 8,000 specific ecosystems, yet widespread environmental degradation persists due to extractive industries and land-use change (196, 197).

The mining and oil sectors play a major role in environmental degradation, contributing 42% and 24% of national pollution, respectively, followed by the waste management sector (14%) (198). At the same time, access to drinking water and sanitation reveals stark inequalities, especially in rural areas and among indigenous and Afro-descendant communities. While 96% of the urban population has access to drinking water, only 70% of rural residents do, with much lower coverage in regions like Chocó and La Guajira (199, 200). Sanitation services show similar disparities, with nearly 4 million rural Colombians lacking safe systems, leading to increased public health risks (201, 202).

These structural challenges result in environmental degradation and economic losses estimated at 1.2% of gross domestic product (GDP), further threatening Colombia’s rich biodiversity and the well-being of vulnerable populations (203, 204). In areas without centralized sanitation infrastructure, decentralized treatment models and non-conventional technologies—such as solar disinfection and membrane filtration—have been proposed as cost-effective alternatives, particularly for improving water quality in underserved communities (205, 206).

Addressing these challenges also requires robust scientific research to guide the implementation of effective bioremediation strategies. Although numerous bioremediation studies have been conducted in Colombia (207, 208), there is a noticeable gap between the amount of research produced and the number of studies published in scientific journals. This disparity is a common phenomenon observed in many developing countries and raises important questions about access to publishing resources and the dissemination of scientific knowledge. Several factors contribute to this issue (209). On the one hand, publishing in open access journals can be financially challenging due to the high fees charged by major scientific journals. On the other hand, there is often a lack of funding to support researchers as they complete or improve their work and in many cases, a broader shortage of resources for research itself and access barriers (210, 211). Developed countries devote between 2% (e.g., France, Portugal, and Canada) and nearly 4% (e.g., the United States, Sweden, and Japan) of their GDP to scientific activities—four to seven times more than what developing countries typically allocate. On average, developing nations invest less than 0.5% of their GDP in research and development; in Colombia’s case, this figure is just 0.29% (212). In contrast, researchers in developed countries—especially those for whom English is a first or second language—benefit from greater access to funding and publication support. By 2020, they accounted for 62% of the articles indexed in the Social Science Citation Index (213). This highlights the global imbalance in research visibility and output between developed and developing nations.

We performed a comparative analysis between scientific production on hydrocarbons and wastewater bioremediation by the country’s leading universities and the number of published papers. A systematic search was conducted in the repositories of undergraduate, master’s, and doctoral theses from the universities with the highest number of publications on the subject. The search criteria included the keywords “bioremediation,” “biodegradation,” “wastewater,” and “hydrocarbons degradation” along with “microalgae, bacteria, and fungi.” The number of research studies performed by universities is shown in Table 4. It is important to note that the search in Universidad Nacional de Colombia repository encountered difficulties, and the results obtained may not fully represent the scientific output for this university.

**TABLE 4:** Colombian thesis on bioremediation of hydrocarbons and wastewater

University	Type of thesis	Number of theses	Repository
Universidad de los Andes	Doctoral	3	https://repositorio.uniandes.edu.co/home
	Master	43	
	Undergraduate	91	
Universidad Nacional de Colombia	Doctoral	0	https://repositorio.unal.edu.co/home
	Master	36	
	Undergraduate	0	
Pontificia Universidad Javeriana	Doctoral	2	https://repository.javeriana.edu.co/
	Master	4	
	Undergraduate	22	
Universidad de Antioquia	Doctoral	0	https://bibliotecadigital.udea.edu.co/home
	Specialization	1	
	Master	1	
	Undergraduate	0	
Universidad del valle	Doctoral	2	https://bibliotecadigital.univalle.edu.co/home
	Master	4	
	Undergraduate	13	
Universidad de Cartagena	Doctoral	2	https://repositorio.unicartagena.edu.co/home
	Master	6	
	Undergraduate	10	
Total		240	

While the survey of research focused on hydrocarbon and wastewater bioremediation yielded 240 studies resulting from undergraduate, master’s, and doctoral theses from various Colombian universities (Table 4), the same search in scientific databases retrieved only 66 published papers (Table S1). From this evidence, we conclude that most of the work related to hydrocarbon bioremediation and wastewater treatment remains unpublished in Colombia. It is important to encourage the publication of scientific work conducted by university students, as publishing scientific papers is one of the main ways for the scientific community to disseminate research findings and to take part in knowledge building (214).

Another concerning trend is the recent decline in scientific publications on hydrocarbon and wastewater bioremediation in Colombia. Although publications increased between 2014 and 2021 (from three to 13 articles per year), a drop has been observed since 2022, with only four, two, and six articles published annually (Table S1). This decrease may reflect reduced government funding for science, limited incentives for student publishing, COVID-19-related disruptions, and a shift in research focus toward other contaminants.

It is necessary to have data that reflects the situation in developing countries in order to determine whether this trend affects only researchers in these regions or also impacts early-career researchers and non-graduate students in advanced economies. However, it was not possible to obtain such data for comparison. This lack of comparative information underscores a structural gap in global research equity, particularly regarding the documentation and visibility of the challenges faced by researchers in developing countries. Bridging this gap is essential for achieving more balanced scientific representation. One effective approach could be to foster stronger collaboration between researchers from developed and developing countries, thereby enhancing inclusive strategies for addressing environmental issues on a global scale (215).

### Microorganisms used for bioremediation studies in Colombia

**Fig 5:**
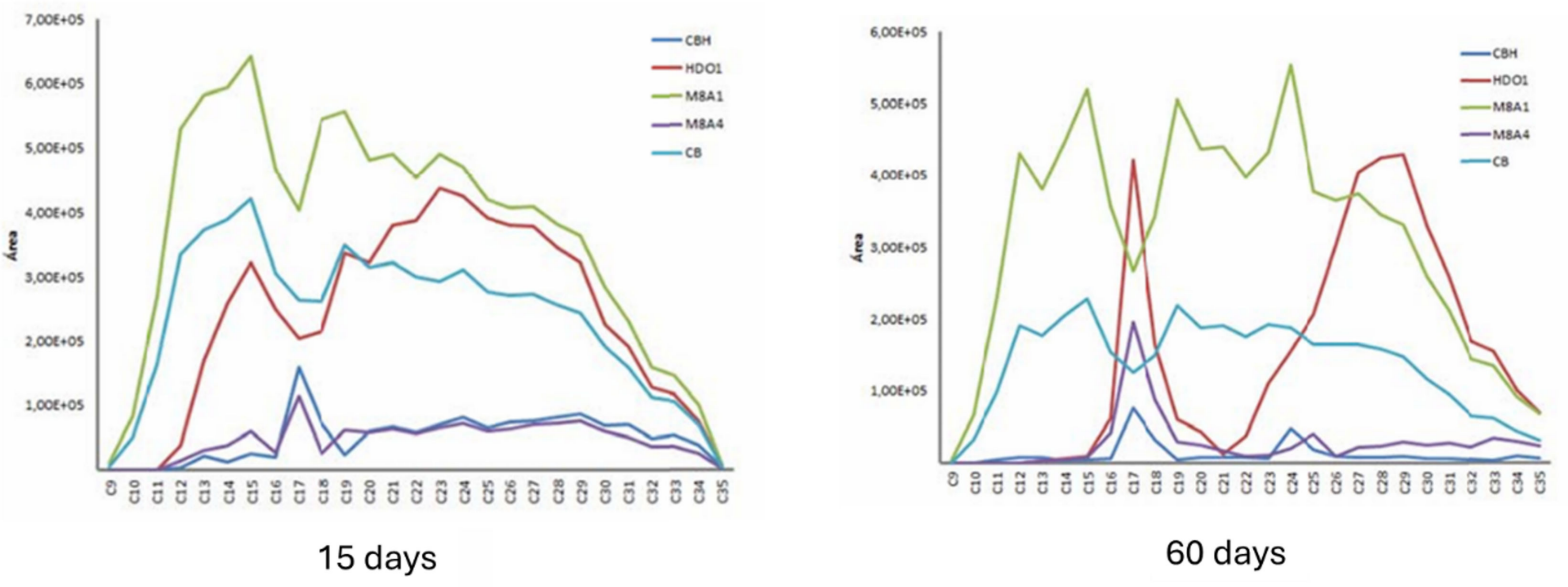
Aliphatic hydrocarbon profile (C9-C35) for *P. aeruginosa* M8A1 and M8A4 and *S. apiospermum* HDO1 individual cultures, consortium CB (M8A1 and M8A4), and consortium CBH (M8A1, M8A4, and HDO1) (from C. J. Sandoval Lozano master thesis, published with permission).

Most published studies on bioremediation focus primarily on bacteria, followed by microalgae, and finally fungi as bioremediation agents (Table S1). Among bacteria, *Bacillus* sp. and *Pseudomonas* sp. are the most commonly studied, while *Chlorella* sp. is the predominant microalga. Our research group previously investigated the degradation of aliphatic hydrocarbons by the fungus *Scedosporium apiospermum* HDO1 and two strains of *Pseudomonas aeruginosa* (M8A1 and M8A4), both individually and in consortium. The results demonstrated that the consortium exhibited superior degradation efficiency, removing 83% of TPHs (Table 5) and achieving 95% degradation efficiency for aliphatic hydrocarbons (Fig. 5) (216, unpublished results).

**TABLE 5:** TPH biodegradation^*a*^^,^^*b*^

Treatments	Days	TPH ppm	Removal (%)	Biodegradation efficiency (%)
Control	15	4,400	NC	NC
	60	2,550	NC	NC
M8A1	15	3,140	28	0.5
	60	2,650	NA	0
M8A4	15	1,170	73	87
	60	2,110	NA	87
CB	15	1,870	58	36
	60	992	61	36
HDO1	15	3,110	29	37
	60	3,090	NA	32
CBH	15	1,680	62	87
	60	428	83	95

^*a*^ Removal percentage = [(TPH control – TPH treatment)/TPH control] × 100. Biodegradation efficiency percentage = 100 – (As × 100/Aac), where As = Area of the peaks in each sample, Aac = Area of the peaks in the abiotic control. NC, not calculated, control treatment. NA, not applicable; the concentration of TPH detected increased with respect to the abiotic control and the previous reading.

^*b*^ Control, negative control without microorganisms. M8A1, *P. aeruginosa* M8A1. M8A4, *P. aeruginosa* M8A4. HDO1, *S. apiospermum* HDO1. CB, *P. aeruginosa* M8A1 and *P. aeruginosa* M8A4. CBH, M8A1, M8A4, and HDO1 (C. J. Sandoval Lozano, unpublished results).

The results of this study demonstrate that although the M8A1 strain lacks the ability to degrade petroleum hydrocarbons, it can synergize with the M8A4 and HDO1 strains to achieve high degradation efficiency. This efficiency surpasses that achieved by these strains individually, both for total hydrocarbons and aliphatic fractions.

### Experiences at pilot scale on hydrocarbon bioremediation

Since 2012, a pilot bioremediation project has been developed at the Caracara oil field in the Los Llanos basin, central Colombia. Molano has implemented an *ex situ* biotreatment process, where oil production sludge is collected and stored before undergoing batch biotreatment. This pilot has tested the bioremediation of soils and fluids contaminated with hydrocarbons at fat and oil concentrations of up to 20 ± 2% by weight, equivalent to 200,000 ± 20,000 ppm (mg carbon/kg soil). By applying biostimulation and bioaugmentation with exogenous bacteria, the process has achieved a removal efficiency of nearly 90% (217).

In 2013, Gutierrez et al. (unpublished data) successfully applied phycoremediation using microalgal consortia to reduce key pollutants in a petrochemical effluent at an industrial production scale. The removal of hydrocarbon contaminants, heavy metals, PAHs, and chlorides from oil field production water proved highly effective (Table 6), with removal percentages ranging from 60% to 90%, similar to those observed in laboratory-scale studies. Conducted at a scale of 30 Kl, this study provided strong evidence of the feasibility of using phycoremediation for large-scale industrial wastewater treatment. Additionally, the process significantly reduced coliform bacteria counts by 99.9% and 97.13%, bringing them down to levels considered non-hazardous (<1,000 NMP/100 mL), even though these microorganisms are not typically found in petrochemical wastewater.

**TABLE 6:** Pollutant removal for two assays from a petrochemical effluent^*a*^

Parameter	Assay no.	Initial concentration (mg/L)	Final concentration M(g/L)	% removal
PAHs	1 2	0.674 0.674	0.001 0.001	99.9 99.9
Chlorides	1 2	840 1,219.62	254.92 247.42	69.7 79.7
Aluminum	1 2	0.318 0.176	0.021 0.021	93.4 88.1
Conductivity	1 2	8,495 9,440	2,700 2,810	68.2 702

^*a*^ μS/cm (unpublished results).

Phycoremediation has been shown to significantly reduce coliform levels in effluents, achieving reductions of approximately 88% (218) and up to 99% in another study utilizing a microalgae consortium, including *Chlorella* sp., *Ankistrodesmus* sp., *Scenedesmus* sp., *Euglena* sp., *Chlamydomonas* sp., *Oscillatoria* sp., *Micractinium* sp., and *Golenkinia* sp. (219). This work also reported that phycoremediation decreased effluent conductivity and heavy metal concentrations by nearly one-third. These simultaneous reductions highlight the potential of microalgae-based (microalgal consortium [MC]) bioremediation as a comprehensive and promising approach for large-scale industrial applications.

In 2014, Manchola and Dussán presented the results of using seven strains belonging to *Lysinibacillus sphaericus* and *Geobacillus* sp. to evaluate their ability to biodegrade TPH in the presence of toxic metals, their potential to produce biosurfactants, and their ability to enhance the biodegradation rate. All seven strains were able to successfully use crude oil hydrocarbons as the sole source of carbon and energy, and their ability to degrade crude oil was not affected by the presence of toxic metals such as chromium and arsenic. At the same time, the strains were able to reduce the concentration of toxic metals through biosorption processes. The production of biosurfactants was also evidenced, which showed an increase in biodegradation efficiency both in liquid minimum salt medium and in landfarming treatments. In the field pilot, a 93% reduction efficiency in TPH concentration was evidenced (220).

In 2015, also in Colombia, a pilot project was carried out to decontaminate soil contaminated by hydrocarbon spills from terrorist actions in the department of Putumayo. Researchers tested a commercial surfactant together with a commercial biostimulant to induce the degradation of pollutants due to the diversity already present in the soil. Researchers reported a 76% degradation of TPH in 60 days (221).

### Experiences at pilot scale on water cleaning in Colombia

Gutierrez et al. (unpublished data) applied phycoremediation to a slaughterhouse effluent to evaluate the ability of a microalgae-based consortium to remove organic waste and bacteria. The raw effluent contained high levels of BOD and COD, as well as extremely elevated *Escherichia coli* counts (exceeding the detection limit of the method used) (Table 7). After treatment with the MC, both organic load and fecal bacteria were effectively removed, making the wastewater safe for discharge into lakes or rivers according to Colombian environmental regulations. Nitrogen content and sulfides were nearly 100% depleted, while fats and TDS were reduced by over 60% and 70%, respectively. These results demonstrate that MC effectively bioremediates common organic contaminants in wastewater at a pilot scale.

**TABLE 7:** Pollutant removal for two assays from a slaughterhouse effluent

Pollutant	Assay no.	Initial concentration (mg/L)	Final concentration mg/L	% removal
BOD	1 2	1,840 3,300	90.3 105	95.1 96.8
COD	1 2	2,638 7,604	263 217.2	90.0 97.1
Total nitrogen	1 2	255.9 559.8	42.4 17.1	83.4 96.9
Fecal coliforms^*a*^	1 2	1.6E+14 1.6E+14	230 225	100 100
Total coliforms^*a*^	1 2	1.6E + 14 1.6E + 14	330 215	100 100

^*a*^ MPN (unpublished results).

In 2018, the 750 hectares Mallorquín swamp was bioremediated with microalgae predominant microbial consortia (MPMC) (222), achieving over 80% removal of its organic load in just 5 weeks of intervention. Initial contamination levels at the swamp exceeded 700 mg/L of BOD5, far above the environmental authority target of 8 mg/L. Those results were the first setting of the effectiveness and ecological safety of MPMC bioaugmentation at that waterbody scale.

In 2019, MPMC bioaugmentation was successfully used to address a cyanobacterial bloom dominated by *Microcystis* sp. and high levels of microcystin, which had caused the death of livestock. Within 15 days, the treatment disrupted the cyanobacteria dominance, restoring the natural phytoplankton diversity. The reduction of *Microcystis* sp. ranged from 34% to 53% in the first 16 days of treatment and reached 100% by day 58. Additionally, MPMC bioaugmentation increased dissolved oxygen levels from 0.3 to 1.8 mg/L, providing evidence of its possible usefulness and safety in controlling cyanobacterial blooms and mitigating cyanotoxin risks for animals and humans (223).

Another successful application of phycoremediation by bioaugmentation with MPMC was observed in 2020 in the lower basin of Arroyo Grande de Corozal, in the department of Sucre, Colombia. In this case, a high removal of the organic contamination parameters coliform bacteria and enterococci and cyanobacteria was achieved both in the stream and in the Santiago Apóstol swamp where it flows into the stream (224).

Recent reports show that hydrocarbons and wastewater phycoremediation is a global trend, driven by the need for sustainable, cost-effective, and eco-friendly solutions to water pollution (225–228).

## CONCLUSIONS AND FUTURE PERSPECTIVES

Bioremediation strategies have been explored for hydrocarbon pollution, and similar approaches are being applied to address the pressing issue of wastewater contamination in megadiverse countries. By using native microorganisms and focusing on more sustainable, nature-based solutions, these countries have the opportunity to reduce the negative environmental impacts of wastewater pollution while also protecting their invaluable biodiversity. Due to the high diversity and innocuity of microalgae compared to bacteria and fungi, our group will continue working with microalgae-based consortia.

International collaboration and funding initiatives will be essential to overcoming current financial and publication barriers, fostering research on low-cost, sustainable bioremediation technologies. Integrating bioremediation into national environmental policies, especially in regions with high biodiversity, and promoting education and training in biotechnology could further drive the advancement of this field. Additionally, empowering rural and local communities by transferring affordable bioremediation technologies could help address pollution challenges at the grassroots level. The future also points to the potential use of synthetic biology and advanced biotechnology to engineer more effective consortia, as well as leveraging emerging technologies like drones and environmental sensors to monitor the success of these efforts in real time.

## SUPPLEMENTAL MATERIAL

Table S1 aem.01442-25-s0001.xlsx Survey of research papers about bioremediation and wastewater treatment in megadiverse countries.

ASM does not own the copyrights to Supplemental Material that may be linked to, or accessed through, an article. The authors have granted ASM a non-exclusive, world-wide license to publish the Supplemental Material files. Please contact the corresponding author directly for reuse.
